# Practices and Perceptions of Community Health Centres Professionals Toward Evaluation: A Qualitative Study

**DOI:** 10.1111/jep.70179

**Published:** 2025-07-31

**Authors:** Madeleine Capiau, David Buetti, Jean Macq, Sophie Thunus

**Affiliations:** ^1^ Institute of Health and Society University Catholic of Louvain Ottignies‐Louvain‐la‐Neuve Belgium; ^2^ Department of Health Management, Evaluation, and Policy, School of Public Health University of Montreal Canada

**Keywords:** care ethics, community health centers, evaluation practices, informal evaluation practices, perceptions of evaluation

## Abstract

**Background and Objective:**

Evaluation plays a critical role in improving the quality and efficiency of services in multidisciplinary primary care organizations, such as community health centers. Despite growing interest in developing tailored evaluation theories for multidisciplinary primary care non‐profit organizations, little is known about how evaluation is practiced and perceived by professionals in community health centers. This paper explores both evaluation practices and professionals' perspectives in Belgian community health centers.

**Methods:**

We conducted semi‐structured interviews with 21 professionals from 12 Belgian community health centers.

**Results:**

The results highlight the complementarity of formal and informal evaluation practices in understanding how evaluation is conducted and used in community health centers. The results highlighted how conflicting considerations regarding relevance, utility, and feasibility that arise in formal evaluation practices lead community health centers to informal evaluation practices. Professionals perceive informal evaluation practices as more aligned with the values of community health centers.

**Conclusion:**

These results highlight the need to adopt a hybrid approach to evaluation, which combines formal and informal practices in a complementary rather than opposing manner. Drawing inspiration from care ethics embedded in informal practices could help reimagine formal evaluation practices in a more human‐centered and context‐sensitive way.

## 1 Introduction

Multidisciplinary primary care has emerged as a cornerstone of healthcare delivery across the globe. This is in response to current challenges within healthcare systems, such as an aging population and the increasing prevalence of multimorbidity [1, 2, 3].

Evaluation is increasingly recognized as a pivotal practice to enhance the quality of activities and services of multidisciplinary primary care, including their efficiency [4, 5, 6, 7, 8]. Driven by both internal expectations and external accountability demands, multidisciplinary primary care organizations are under growing pressure to evaluate the value of their services and activities [9, 10, 11].

The growing interest in evaluating the quality of activities and services has led to numerous theories of evaluation practices, highlighting different ways of conducting and using evaluation [12]. These theories guide stakeholders such as evaluators, public authorities and professionals in generating knowledge about the value of their activities and services through varied approaches and methods [13, 14, 15, 16].

Despite growing interest in the development of tailored evaluation theories for multidisciplinary primary care non‐profit organizations [17, 18], little is known about how evaluation is practiced in such setting [19, 20, 21], and even less about how professionals perceive these practices [19, 20, 22]. To date, previous research has focused on evaluation practices and attitudes of professionals in non‐healthcare non‐profit organizations [23, 24, 25, 26, 27]. When literature exists in healthcare organizations, it examines the perceptions of public authorities and evaluators, without considering the perspectives of professionals [28, 29, 30]. However, professionals are most often the primary intended users of evaluation, defined as the stakeholders most closely associated with the program who are potentially in a position to make decisions based upon the decisions [31].

Understanding the perspectives of professionals as primary intended users of evaluation provides a grounded understanding of how evaluation is really experienced, which can differ significantly from how evaluation is theorized. By integrating professionals' perspectives, it becomes possible to identify ways to improve evaluation practices in multidisciplinary non‐profit primary care organizations, not simply as a mandatory obligation for public authorities, but as a fundamental and meaningful component of organizational learning and continuous improvement [24, 32, 33, 34, 35]. Moreover, understanding how professionals practice and perceive evaluation can inform targeted efforts to build evaluation capacity strategies [36].

Community health centers [CHCs] are among the institutions that embody such an approach of multidisciplinary primary care. They are non‐profit organizations that aim to respond to the health and care needs of individuals within their local communities. Their mission is to deliver comprehensive, integrated, and continuous support [37]. As increasingly important multidisciplinary primary care organizations in Belgium, CHCs are concerned with evaluating their activities and services. However, to date, little is known about how evaluation is conducted and used within these organizations, particularly from the perspective of professionals.

Our paper addresses these gaps by exploring both the evaluation practices in Belgian community health centers and the social representations of these practices among professionals. Social representations are defined as the set of characteristics and properties that individuals (i.e. the various stakeholders involved in the evaluation) attribute to an object of representation (i.e. the evaluation) [38]. In this way, social representations, as systems of values and beliefs about the object of representation, make it possible to situate the attitudes and behaviors of individuals with this object. Studying professionals' representations of evaluation thus provides insight into how they understand and shape evaluation practices. More specifically, our paper addresses the following two research questions: (i) What do evaluation practices look like in Belgian community health centers, in terms of conducting and using evaluation? (ii) How do CHCs professionals perceive the relevance, utility, and feasibility of these evaluation practices?

## 2 Methods

### 2.1 Research Design

To explore the practices and representations of evaluation among Belgian CHCs professionals, we conducted a qualitative study using an exploratory and interpretivist approach, employing semi‐structured interviews.

### 2.2 Setting

We chose to focus on Belgian CHCs affiliated with the Federation of Community Health Centers [FCHC]. In 2025, there are 138 CHCs in Wallonia and Brussels affiliated with this Federation, employing nearly 2800 professionals and providing care for approximately 300,000 patients. There are approximately 11 full‐time equivalents per CHC, comprising multidisciplinary profiles [39]. Each CHC can choose between two models of payment: fee‐for‐service and capitation, whereby the National Institute for Health and Disability Insurance pays the CHCs a fixed contribution every month per registered patient. The CHCs register patients and, through their multidisciplinary teams, provide them with preventive and curative care without a personal financial contribution at the point of contact [40]. Members of the FCHC must adhere to a set of criteria that includes values, targets, and methods outlined in a charter [41].

Although there are many CHCs in Belgium outside this Federation [42], those affiliated with it align most closely with the integrated community primary care model promoted by the World Health Organization [42]. Furthermore, affiliated CHCs with this Federation represent approximately half of all Belgian multidisciplinary primary care organizations affiliated with a federation, making them a relevant and representative sample for understanding broader trends and challenges in all CHCs.

### 2.3 Participants

We carried out in‐depth qualitative interviews with 21 professionals from 12 CHCs, which are based in Wallonia and Brussels. Given that this study explores practices and representations of evaluation, all professionals in CHCs were eligible for this study. We specifically interviewed four nurses, seven physicians, one physician assistant, one physiotherapist, one psychologist, four coordinators, and three receptionists.

### 2.4 Data Collection

Professionals of 22 CHCs received a written description of the project by email. These 22 CHCs were chosen for their diverse organizational characteristics (model of payment, age and team size). 12 CHCs were contacted and provided with contact information for one of their professionals. These 12 professionals helped us recruit an additional 9 participants for the study through snowball sampling.

The interviews were conducted between September 2021 and June 2022. Half of the interviews took place at the institutions where the professionals worked. The other half was conducted via videoconferencing using *Zoom* and *Microsoft Teams*. Professionals from the same CHC were interviewed separately to allow space for diverse perspectives on evaluation. The same researcher conducted all the interviews. Interviews were conducted in French and lasted approximately 1 h.

An interview topic guide was developed and iteratively adjusted to incorporate emerging perspectives. The interview guide explored three main topics: (i) evaluation practices, (ii) attitudes toward these evaluation practices, and (iii) barriers and enablers that have been encountered when practicing evaluation. In this paper, we focus on the first two topics. To gather comprehensive insights into representations of evaluation, we have opted not to explicitly define the term ‘evaluation’ in the interview topic guide. Furthermore, we also did not focus on a specific data collection method or approach related to evaluation practice. We have only stipulated that the interview's questions encompass current and past evaluation practices, whether they occur at an individual, collective, or organizational level.

### 2.5 Data Analysis

All the interviews were transcribed. The content analysis of the data collected from our interviews was conducted using inductive coding, using NVivo 12 qualitative data management software. The first author became acquainted with the texts by reading all the transcripts multiple times. Notes were taken, and preliminary codes were directly derived from the data. The set of codes was discussed with two senior researchers, who analyzed some of the texts. The interview excerpts used in this paper were translated from French to English during the writing process, accompanied by checks to maintain the integrity and intended meaning of participants' responses.

### 2.6 Ethics

Ethical approval was obtained from the Hospital Departmental Ethics Committee of Saint‐Luc, Catholic University of Louvain, in Brussels (ID: 2021/17MAI/225). The participants were informed of the study's purpose both verbally and in writing, and verbal consent was obtained for audio recording. All participants were offered anonymity and informed that identifiable information would not be submitted to any third parties. In the results, participants are identified by their professional profile and the order in which they were interviewed.

## 3 Results

In questioning CHCs professionals about what they do in terms of evaluation, we highlighted two distinct types of practices, which we called ‘formal’ and ‘informal’ evaluation practices.

We have chosen to use the label ‘formal evaluations practices’ for activities that professionals explicitly and univocally identify as evaluative. In contrast, we applied the label ‘informal evaluation practices’ to activities that professionals are uncertain whether to classify as evaluative due to their distinct design, which deviates from conventional evaluative forms. This differentiation enables us to account for a broader range of evaluative activities, including those that may not conform to traditional criteria but still contribute to the evaluative process in a less structured manner.

We begin by exploring the distinctive characteristics of the so‐called ‘formal’ evaluation and then go on to present the manner in which formal evaluation practices came into tension with certain considerations of CHCs. Then, we present how these conflicting considerations in formal evaluation practices lead professionals to rely on strategies, such as informal evaluation practices, and conclude by presenting how professionals view them.

### 3.1 Conducting and Using Formal Evaluation Practices

First, CHCs are required by public authorities to complete administrative reports. These reports exclusively compile quantitative data, such as the number of people served by their activities and services, budget and expenditures: “*In terms of evaluation, we already have the community health forms, since every year we have to fill in these forms for the INAMI. The data to be supplied must be almost exclusively quantitative*” (Nurse 3). To support these reports, they primarily rely on their monitoring activities, in which they track the demographic characteristics of registered individuals.

Second, professionals report relying on formal evaluation programs that are largely pre‐designed and implemented as turnkey solutions to assess the quality of their services. These programs are theoretically based on participatory self‐evaluation, employing a qualitative approach, with the occasional assistance of a facilitator. Some of these programs are mandated by funders, while others, such as the *DEQuaP* program, specifically developed by the Federation for CHCs, are offered as optional tools for evaluation [42, 43]. In the second case, teams used them because they were struggling to do an evaluation due to their lack of resources and thought they might be useful to start an evaluation process. They also hoped these turnkey programs, based on an internal and participatory approach, could be guided by the teams themselves, as participation is a core value of CHC. They also believed these qualitative‐based approach turnkey programs could help explore the subjective experience of work and relationships, which fits with CHCs' values.

The fact that DEQuaP is a process, that is turnkey, can really encourage the team to engage with it.

(Receptionist 2)

As physicians, we have no training in how to evaluate by ourselves, so we were hoping that the program would provide us with some answers.

(Physician 2)

### 3.2 Perceptions of Formal Evaluation Practices

Administrative reports required of CHCs are not perceived as effective activities for learning and improvement. Professionals report that these reports, primarily based on quantitative data, fail to capture the complex and nuanced nature of everyday practice, and as a result, tend to offer an overly simplistic portrayal of the realities within CHCs. Moreover, quantitative data seem to be too limited to answer the question “where do we go from here?”. As a result, professionals find it challenging to effectively utilize these indicators in ways that support a deeper inquiry into the quality and impact of their work.

These evaluation processes for care professions are based on indicators, but I find them a little disembodied. For example, knowing that we had 20000 consultations in 1 year compared with 17000 the year before, I don't see what that adds up to. It gives a vague idea of the workload, but I found it too abstract.

(Nurse 4)

We have activity reports to submit, but I know that in our CHC, we try to keep the figures as unimportant as possible because I found that they provide a rather watered‐down vision of what a primary care organization should be. Our team struggles with quantitative data because we look after people.

(Receptionist 2)

Professionals, therefore, only see them as a compliance measure that is more useful to those requesting the evaluation than to those implementing the evaluated activity, e.g. CHCs professionals:

I'm thinking typically of the reports you have to do sometimes after the subsidies, there's paperwork. I have to fill it in because it's compulsory, but when I've finished the assessment, it hasn't done me any good. It doesn't make sense to me!

(Physiotherapist 1)

By imposing this type of data and evaluation, professionals report the risk of filling the administrative reports in a way that presents them in the best possible light due to the stakes of subsidiaries: “*The goal is to get the subsidy, and then you'll have to hustle to justify your subsidy*” (Physiotherapist 1).

Moreover, while professionals used turnkey evaluation programs expecting their approach could fit with their values of CHCs and could be useful to enhance the utilization of evaluation process and results for quality improvement, they also expressed disappointment after investing significant amounts of their already stretched working time in these turnkey evaluation programs. They only observed minimal changes to their practices: “*There was just one thing with this evaluation program, and that was that we were very disappointed with the results. For a while, we had the impression that we'd done it all for nothing*” (Physician Assistant).

First, even if the programs are based on a qualitative approach, professionals complain that evaluation criteria are still predetermined outside the CHC. Consequently, they do not feel that they are discussing what truly interests them regarding their realities and needs; they are confined to what is proposed in the evaluation program, which is not always consistent with the practices or contextual realities of their CHCs: “*I had the impression that we were getting bogged down in all these processes when it's something very theoretical and which should work, but which doesn't take into account the nuances of the field*” (Nurse 1).

Second, professionals highlighted that they did not feel genuinely included as key actors in the evaluation process but rather felt like passive participants. The team's lack of a sense of ownership of the process had a significant negative impact on the utilization of the evaluation results: “*I don't think it has done us any good. We were more inclined to botch the subjects or the information we were given, and in the end, I confess I didn't even retain any of it*” (Physician 2).

Third, in the case of an external facilitator was there, professionals think he would enable a discursive and participatory approach in line with their values. They didn't, however, view the facilitator as a learning resource because she didn't allocate sufficient time to work with them in a participatory and bottom‐up manner. Professionals believe that this difficulty is related to the external profile of the facilitator. They observed that the facilitator did not understand CHC values and was therefore unable to capture the essence of the CHC

She [the facilitator] regulated the time but didn't necessarily help us well to understand and explore the questions in depth.

(Physician Assistant)

We bring someone in from outside, but many believe outsiders are unfamiliar with the movement and thus incapable of understanding it.

(Physician 7)

### 3.3 Strategies to Resolve the Considerations of Formal Evaluation Practices: Conducting and Using Informal Evaluation Practices

The conflicting considerations that arise in both formal evaluation practices lead professionals of CHCs to rely on different kinds of strategies.

On the one hand, because they have to fill administrative reports, a common strategy used by teams to “make it work” is to streamline the required evaluation by assigning it to just one individual or to a subset of the team. Instead of being a collective and discursive process, evaluation becomes a rapid and mechanistic procedure, lacking reflective thought and growth. Some professionals, however, are uncomfortable with this way of doing things because the multidisciplinary approach, along with participatory and collective values, is inherent to CHCs.

It's often one or two people who take care of the mandatory processes, which are figures to be handed in to the administration: there's no collective ownership of the process. More and more, it was the responsibility of the person coordinating health promotion, and I felt that the rest of the team was losing interest in this aspect.

(Nurse 4)

On the other hand, professionals report conducting formal evaluation practices only when required by funders. They sometimes delegitimize these formal evaluation practices to rely on informal evaluation practices instead. Indeed, professionals explicitly stated that they are engaged in other evaluation activities besides formal ones. Some professionals labeled these evaluation activities as ‘informal’: “*The difference between formal and informal is important, there really is a lot more informal than formal evaluation*” (Coordinator 3).

They call these ‘informal’ in contrast to formal evaluation activities, with a systematized and formalized nature. When asked to describe more what they considered ‘informal’ evaluation activities to be, they primarily associate them not so much with the degree of formalization but with the underlying process of reflection, regardless of the methodology used: “*There was no formal evaluation, but we participated in meetings on the dashboard, extracted data, and thus we reviewed our practices*” (Nurse 4). Nonetheless, these activities are more difficult to recognize because they are far removed from formal evaluation activities. Yet, some professionals are unsure how these activities could be included in the scope of evaluation without being formalized by a final report or using a scoring approach.

There's a topic under discussion, and everyone shares their point of view, or there are people who have done research. But is it an evaluation without a final written product?

(Physician 3)

It's not an evaluation because we are not using a system where a grade is assigned (…) We ideally should also draw up reports to compile the results of our evaluations and decisions.

(Nurse 3)

The common element in all informal evaluation activities is deliberative dialog between professionals, sometimes with no product such as a report: *“Generally speaking, we do things orally. Team meetings are therefore recorded in writing, but most of the time things are clearly done orally”* (Physician Assistant). The data used in these informal evaluation activities is based much more on experiences and subjectivity, with the expression of feelings being central. A common tool used in these peer‐group discussions is storytelling, whereby professionals develop their critical reflection skills by thinking about challenges and ways to address them and improve. Specifically, exchanges relate to clinical care work and involve unpacking problems and formulating solutions for real‐time uptake. Professionals raise challenges they face in their practice and ask their colleagues for ideas:

We didn't use an evaluation grid, but focused more on the feelings, saying ‘this was good, this is much better.

(Nurse 3)

We carry out intra‐sector evaluation processes, and I know that in the nursing sector we try to do them regularly. We regularly set aside moments to take a critical look at our practices, which is still interesting. We discuss the patient's situation. Then we analyze how it went and what we were able to put in place.

(Nurse 3)

When I have complicated cases for example, we get together with the nurse and try to say: ‘Well, what could we have done’ or ‘what should we do?’ I do the same with the social worker and the physician.

(Physician Assistant)

Informal evaluation practices are usually the result of personal initiatives, unplanned, and take the form of “*corridor discussions*” (Nurse 1) among a few professionals. They occur in the kitchen, in the hall, or around the coffee machine: *“In our team, we're very pro‐informal. The more we talk over a cup of coffee, the better”* (Nurse 1). Other informal exchanges are scheduled and slightly more structured, such as organized meetings involving all staff or a select few.

The use of informal evaluation activities can, therefore, be attributed to the manifold advantages they offer to the professionals interviewed, in contrast with the utility, legitimacy and feasibility considerations of formal evaluation practices.

Informal evaluation activities are embedded in everyday practice rather than being a time‐consuming add‐on like formal ones. They facilitate real‐time feedback without creating an additional burden that might compromise patient care: “*We try to test several different things, but small things that can be done quickly because we have constant emergencies, so informal practices are a bit better suited to our needs, given the amount of time we have*” (Receptionist 2).

Such informal evaluation activities are discursive in nature. Free exchanges during these activities make professionals feel much more involved in the evaluation process, whereas some formal evaluation activities, though supposedly participatory, do not. Indeed, informal evaluation practices provide opportunities for professionals to engage in reflexive and genuine discussions that are relevant to their everyday practices, without being detrimental to their care work: “*We need to make sense of things even if we think we don't have the time*” (Nurse 4). Dialog also allows them to fully acknowledge the inherently subjective nature of their work, which they find is made difficult by the predominantly quantitative nature of most formal evaluation practices. Moreover, according to professionals, these informal evaluation practices provide a safe space where they can discuss their difficulties, thereby strengthening their ability to be understood.

There is indeed a very qualitative side to the way we provide care; it is more difficult to measure quantitatively because it is more subjective.

(Receptionist 2)

We talk a lot about what we're going through and the difficulties we're experiencing. We take everyone's difficulties into account. And if we don't understand them, we ask ‘Is this something you can do at reception? Do you have this or that difficulty?’.

(Physician Assistant)

The discursive approach inherent to informal evaluation practices also has advantages in relation to team dynamics. It strengthens collaboration by enabling professionals to better understand each other and their differences. Moreover, informal everyday practices are a good way to recognize work that has been accomplished and the challenges of day‐to‐day clinical work.

We don't all have the same vision, and that has been a driving force in moving forward because it's the richness of everyone's thinking that makes it all possible.

(Physician 2)

Speaking subjectively gives us the flavor we need (…) We've had suicides among patients, it's not easy to handle. When something goes well, we need to celebrate it. We like to celebrate it, to say it, to encourage ourselves, to see what's beautiful right away, otherwise we'll get depressed.

(Physician 5)

## 4 Discussion

This qualitative exploratory study examines how Belgian professionals in community health centers practice and perceive evaluation, with the aim of providing more relevant, practical, and feasible evaluation theories tailored for these organizations and their primary intended users, the professionals [35, 44].

In response to our first question regarding the evaluation practices in CHCs, our results highlighted that professionals conducted both formal and informal evaluation practices. By examining the different activities mentioned by the professionals interviewed, we were able to identify several distinctive characteristics they attribute to each evaluation activity.

These distinctive characteristics align with the distinctions already proposed in the spectrum of evaluation discussed by various authors [23, 24, 45, 46, 47, 48, 49, 50]. At one end of the spectrum, formal evaluation practices are characterized by a systematic and rigorous approach, involving sequential phases of data collection, analysis, and reporting, with the goal of producing an evaluative report. Professionals also characterize these formal evaluation practices by their use of objective data and data sources [45]. In terms of data, objectivity is defined by the professionals as relying primarily on quantitative indicators derived from measurement tools that are also considered objective in nature, such as dashboards. On the contrary, according to the literature [23, 24, 45, 46, 47, 48, 49, 50], informal evaluation practices include activities that involve processes of reflective and interpretive judgment, taking into account quality, value, significance, and merit, regardless of whether they are procedural or scientific in nature. Subjectivity was argued on the basis of the type of data used, such as experiences, feelings, and lived experiences of the professionals during oral exchanges. This perception holds that the main design of the informal evaluations is based on a discursive approach [51, 52]. In practice, we saw that this definition encompasses a range of informal activities, such as qualitative feedback, informal reflections, and day‐to‐day observations from professionals, which are integrated into the daily practice of organizations. These processes and results are rarely documented in a final report [50]. Between those, a large space exists with a variety of approaches [13, 53].

In this paper, we choose to call ‘formal’ and ‘informal’ practices not simply for the way professionals are conducting evaluation, but also to reflect the representations of professionals regarding evaluation activities. We labelled some of evaluation activities as ‘formal’ because professionals unequivocally classified them as evaluation. Conversely, we have categorized some evaluation activities as ‘informal’ because professionals are uncertain whether they qualify them as evaluation due to their differences with the accepted representation of ‘formal’ evaluation activities.

The fact that professionals themselves are hesitant or do not always recognize informal activities as evaluation shows the dominance of a social and political vision of rational and structural evaluation practice [9, 23, 49, 51, 54, 55, 56]. The literature shows that the most formal evaluation practices still dominate the evaluation landscape of healthcare organizations, as evidenced by the increasing number of accreditation programs [9, 49, 51, 54, 55].

Unlike other healthcare organizations, our results suggest that evaluation activities in CHCs predominantly follow internal and participatory approaches, situating them midway between formal and informal evaluation practices. However, in response to our second research question, our results show how professionals display hesitation regarding the specific characteristics of these formal evaluation practices and its impact on its use. Indeed, evaluation activities labelled ‘participatory’ cannot keep their positivist promises or achieve their objectives if participation and empowerment are artificial [30, 48]. Whilst participatory evaluation literature suggests that it contributes positively to evaluation by enhancing the utilization of evaluation results, the possible negative effects have been less discussed [57]. Yet, our results show that evaluation program designs theoretically based on a participatory approach (e.g., *DEQuaP*) encountered negative reactions, such as frustration due to the loss of time and financial resources without any discernible changes. Consequently, this evaluation activity was viewed as a pseudo‐evaluation rather than an active process capable of producing evaluative knowledge and facilitating a shift from conventional, rational and objective evaluation practices to more inclusive ones.

Regarding administrative reports described as evaluation practice by our participants [4, 23], they criticize them because they are often being imposed by public authorities. This entails an adoption of evaluation by the organization merely as a tool for accountability [4, 20, 23], and most of the time evaluation practice is delegate to one or two members of the team. This lack of collective ownership does not incorporate the values of participation and empowerment, which are central to the values of CHCs. The evaluation process is often imposed and relies on externally driven indicators. These indicators are described by our participants as overlooking important contextual and human factors. Moreover, the indicators used are mostly quantitative and outcomes‐oriented.

Although the necessity of distinguishing accountability from evaluation [4, 23] is acknowledged, the limited perceived contribution of administrative reports to learning raises important questions about how to reconcile public requirements with professionals' learning needs, especially given their limited resources [21]. Our results highlighted the resistance to these quantitative indicators [19, 20]. However, quantitative indicators are not an end in themselves. They can provide food for thought regarding how to improve the quality of CHCs' services. They should be used as a starting point for developing the reflexivity of professionals [58].

One the contrary, informal evaluation practices have been documented within non‐profit organizations as much more relevant and effective for learning and quality improvement purposes in contrast to the low utility of the most formal evaluation activities [23, 24, 48, 49, 50]. Such forms of informal activities reported in our results are in line with Schön's work on reflective practice, including reflection‐in‐action and reflection‐on‐action [59]. They have always existed but seem to be increased ‐ or at least better documented ‐ in non‐profit organizations [23, 24, 48, 49, 50].

The positive perceived helpfulness and relevance of informal evaluation practices, compared to formal ones, provide valuable insight into what primary intended users of evaluation consider appropriate for CHCs. These perceptions prompt us to reflect on what informal evaluation activities can contribute to formal ones.

By reclaiming a space for sharing their experiences, emotions, and feelings through informal evaluation practices, professionals move outside the norm of rational and objective evaluation practices without negating the importance of evaluation. They create a ‘caring’ space for evaluation where knowledge can be valorized. Consequently, professionals underscore the importance of a truly participative approach in formal evaluation activities, aligning with key values of ownership, meaningfulness, transparency, mutual benefit, and collaboration of CHCs [48, 51].

The ‘caring’ space created by informal evaluation practices can be linked to the perspective of *care ethics* [51, 60, 61, 62, 63]. As Visse & Abma [64, p. 5] explained: “*care ethics generally moves from what is just, from rights and principles to what matters to people, to ass the import of things for people, their evaluative judgments. It also moves from reason to perception and experience: putting lived experiences in everyday situations at the center of attention*”. Some evaluation approaches are grounded in insights similar to values from care ethics. Examples of these approaches include democratic evaluation, responsive evaluation [65], and transformative evaluation. They are all aimed at applying more participatory and democratic approaches to evaluation. We, therefore, suggest that the current participatory evaluation approaches used in CHCs could be further enriched by drawing inspiration from the principles of *care ethics*, as currently suggested by many authors [51, 66, 67, 68]. By incorporating principles from *care ethics*, formal evaluation activities can not only improve the quality of the services offered but also enhance the value of professionals, which represents a major current managerial challenge [69]. Building on existing studies conducted in other contexts [51, 68, 70, 71], future research should explore how *care ethics* can be applied to evaluation practices within multidisciplinary primary care organizations, such as CHCs.

Our study has strengths. First, its qualitative approach gives voice to Belgian CHCs professionals, capturing their perspectives and experiences with evaluation. Second, to our knowledge, it is the first study to specifically explore evaluation practices within these multidisciplinary primary care organizations. As such, it fills an important gap in literature and offers interesting future research in this area. Our study also has some limitations. It was exploratory and involved only 21 professionals from 12 CHCs, which may not capture the full diversity of views on evaluation practices. Future research could examine different subgroups in greater depth and explore additional topics regarding evaluation, such as professionals' perspectives on patient involvement and evaluation responsibility. Expanding the scope to include more CHCs and using methods like observations and surveys could also highlight a wider range of formal and informal evaluation practices.

## 5 Conclusion

By examining the practices and perceptions of professionals from Belgian CHCs, this study contributes to ongoing reflections on how evaluation practices can be improved to enhance their relevance, utility, and feasibility for primary intended users. In doing so, it supports a shift toward evaluation approaches that better promote learning and the improvement of care quality. Our results highlighted the value of informal and participatory evaluation practices, suggesting that *care ethics* may offer a meaningful lens for rethinking evaluation in CHCs, as well as in other multidisciplinary primary care organizations.

## Ethical Considerations and Consent to Participate

The study was approved by the Ethics Committee of Louvain Catholic University Hospital. The consent of the professionals interviewed for this study was given verbally at the beginning of the interviews, before and after the start of the audio recording.

## Conflicts of Interest

The authors declare no conflicts of interest.

## Data Availability

The data that support the findings of this study are available from the corresponding author upon reasonable request.
